# Determinants of antenatal and delivery care utilization in Tigray region, Ethiopia: a cross-sectional study

**DOI:** 10.1186/1475-9276-12-30

**Published:** 2013-05-14

**Authors:** Yalem Tsegay, Tesfay Gebrehiwot, Isabel Goicolea, Kerstin Edin, Hailemariam Lemma, Miguel San Sebastian

**Affiliations:** 1 Deputy head for Tigray Regional Health Bureau, Mekelle, Ethiopia; 2 Department of Public Health, College of Health sciences, Mekelle University, Mekelle, Ethiopia; 3 Department of Public Health and clinical Medicine, Umea University, Umea, Sweden; 4 Department of Nursing, Umea University, Umea, Sweden

## Abstract

**Introduction:**

Despite the international emphasis in the last few years on the need to address the unmet health needs of pregnant women and children, progress in reducing maternal mortality has been slow. This is particularly worrying in sub-Saharan Africa where over 162,000 women still die each year during pregnancy and childbirth, most of them because of the lack of access to skilled delivery attendance and emergency care. With a maternal mortality ratio of 673 per 100,000 live births and 19,000 maternal deaths annually, Ethiopia is a major contributor to the worldwide death toll of mothers. While some studies have looked at different risk factors for antenatal care (ANC) and delivery service utilisation in the country, information coming from community-based studies related to the Health Extension Programme (HEP) in rural areas is limited. This study aims to determine the prevalence of maternal health care utilisation and explore its determinants among rural women aged 15–49 years in Tigray, Ethiopia.

**Methods:**

The study was a community-based cross-sectional survey using a structured questionnaire. A cluster sampling technique was used to select women who had given birth at least once in the five years prior to the survey period. Univariable and multivariable logistic regression analyses were carried out to elicit the impact of each factor on ANC and institutional delivery service utilisation.

**Results:**

The response rate was 99% (n=1113). The mean age of the participants was 30.4 years. The proportion of women who received ANC for their recent births was 54%; only 46 (4.1%) of women gave birth at a health facility. Factors associated with ANC utilisation were marital status, education, proximity of health facility to the village, and husband’s occupation, while use of institutional delivery was mainly associated with parity, education, having received ANC advice, a history of difficult/prolonged labour, and husbands’ occupation.

**Conclusions:**

A relatively acceptable utilisation of ANC services but extremely low institutional delivery was observed. Classical socio-demographic factors were associated with both ANC and institutional delivery attendance. ANC advice can contribute to increase institutional delivery use. Different aspects of HEP need to be strengthened to improve maternal health in Tigray.

## Background

Despite the international emphasis in the last few years on the need to address the unmet health needs of pregnant women and children, progress in reducing maternal mortality has been slow. This is particularly worrying in sub-Saharan Africa where over 162,000 women still die each year during pregnancy and childbirth, most of them because of the lack of access to skilled delivery attendance and emergency care [1-5].

Numerous studies have established the inverse relationship between skilled attendance at birth and the occurrence of maternal deaths. Thus, the significant variation in maternal mortality estimates between different localities within the same region can be attributed, to a large degree, to differences in the availability of and access to modern maternal health services [3,6]. According to the World Health Organisation (WHO), 60 million deliveries take place worldwide each year in which the woman is cared for only by a family member, by an untrained traditional birth attendant, or by no one at all [7].

Low use of maternal health services for delivery has long been on the research agenda. Being a long distance from health services, high costs, multiple demands for women’s time, low coverage and poor quality of care have been identified as key factors [7,8].

The lack of decision-making power of women within the family and inequities in the provision of essential maternal health care interventions remain a challenge in many sub-Saharan African countries [5,9]. Moreover, gender discrimination and low levels of female education prevent women from seeking care, and accessing the best choices for themselves and their children’s health, resulting in critical delays and unnecessary complications and deaths [5,10]. Along with these factors, the relative contribution of antenatal care (ANC) to maternal health has been debated. [11,12]. However, a systematic review revealed a positive association between care during pregnancy and the use of safe delivery services [13].

In order to decrease maternal morbidity and mortality, strong health systems offering accessible, available and satisfactory care are needed. This includes family planning and safe abortions as well as ANC, skilled delivery and postpartum care. All of the reproductive health services should be connected to responsive and accountable emergency obstetric services for the purposes of consultation, transportation and referral [14,15].

### Maternal health care in Ethiopia

With a maternal mortality ratio of 673 per 100,000 live births and 19,000 maternal deaths annually, Ethiopia is a major contributor to the worldwide death toll of mothers. Although improvements have been reported in regard to reducing infant and child mortality in the country, there has been slow progress regarding Millennium Development Goal 5 (MDG5) , the cornerstone of maternal health [16].

In order to improve accessibility to family planning, safe abortions, ANC, skilled delivery and postpartum care, the Ethiopian Ministry of Health has launched a community-based

Health-care system in 2003, the Health Extension Programme (HEP), which is rooted in the primary health care approach. The HEP is designed to improve equitable access to preventive essential health interventions through community-based health services and to achieve significant basic health-care coverage in the country through the provision of a staffed health post serving approximately 5,000 people in a sub-district (*tabia*). Each health post is staffed by two female health extension workers (HEWs) who are assigned after completing one year of training. The HEWs deliver health care services both at the health post and in the community, with a strong focus on sustained preventive health actions and increased health awareness. The HEP has been implemented throughout Ethiopia, with more than 33,000 HEWs already trained and deployed since 2004 [17]. The HEP in rural areas is composed of 16 packages of interventions categorised into four areas: ‘hygiene and environmental sanitation’ (seven packages), ‘family health’ (five packages), ‘disease prevention and control’ (three packages), and ‘health education and communication’ (one package).

With regard to maternal health, HEWs are expected to provide post-abortion care, family planning, ANC, clean delivery attendance and postnatal care. Furthermore, they are responsible for referring women with obstetric complications to health centres and hospitals where basic and comprehensive emergency obstetric care is available [18]. HEWs are also in charge of supervising traditional birth attendants (TBAs) and other voluntary community health workers who are expected to support health education within communities [10,18,19]. Despite this enormous effort, universal access to maternal health services remains limited, particularly when it comes to skilled delivery attendance. The Ethiopian Demographic and Health Survey (EDHS) 2011 reported that 29% of women were using modern contraceptives, 34% were attending ANC, 10% were receiving skilled delivery assistance and 9% postnatal care (PNC) [20]. Although these figures are low, these data reflect improvements compared to the situation in 2005.

In Ethiopia, women’s sociodemographic characteristics – such as marital status, education, parity, access to health services and economic status ‒ have been identified as important factors positively associated with skilled delivery attendance. Moreover, the lack of working time by HEWs for antenatal and delivery care and decisions made by husbands and elderly parents were found to be other important determinants [21-26]. It has also been reported that institutional delivery service and postnatal care service utilisation is still low in the health facilities compared with services provided by TBAs. However, information from community-based studies in rural areas of the country is limited. Very few community based studies of these issues have been conducted in Ethiopia, and none in Tigray. Therefore, this study aims to determine the prevalence of maternal health care utilisation and explore its determinants among women aged 15–49 years living in rural areas in the northern region of Tigray, Ethiopia. By elucidating the determinants, our goal is to provide suggestions for better implementation of maternal health-care services in this setting.

## Methods

### Study area

Tigray regional state is located in the northern part of the country and has an estimated total population of 4.3 million of which 50.8% are females. Eighty percent of the population are estimated to live in rural areas and the majority of the inhabitants are Christian [27]. The region is divided into seven zones and 47 *weredas* (districts), of which 35 are rural and 12 are urban. There is one specialised referral hospital as well as five zonal hospitals, seven district hospitals, 208 health centres and more than 600 health posts in the region. Coverage estimations from the Tigray Health Bureau indicate 75% are for ANC, 20% for skilled delivery, 13% for clean and safe deliveries (those attended by HEWs) and 90% for contraceptive use [28].

The study district of Samre-Saharti is located in the northern part of the state of Tigray, 55 km from the capital, Mekelle. The district has 23 *tabias* (sub-districts) and each *tabia* has a health post with two HEWs. There is one health centre (HC) in the district’s town which functions as a referral center to the four HCs stationed in the rural areas. Samre-Saharti has an estimated population of 124,499 of which 50.2% are female [26]. Women of reproductive age (15–49 years) constitute approximately 14,375 (23%) of the population and the number of deliveries in 2007 was estimated to be 646 [27].

### Study design and sampling

The study design was a community-based cross-sectional survey. Of the 23 *tabias* in the district, four were difficult to reach due to floods and were therefore excluded, so 19 were included in the study. A lottery was drawn among the 19 *tabias* to sort their respective villages (*kushets*) randomly and then the cluster sampling technique was used to select the study population.

The sample size for the study was determined using the single population proportion formula. Assuming the proportion of institutional delivery to be 6%, a 95% level of confidence, a 2% marginal error and a design effect of 2, and the required sample size was 1,115 women.

The 30 clusters were chosen by population proportional to size sampling. Households were chosen after a random start at a central place in the village. A pen was spun and the data collectors walked to the edge of the village in the direction that the pen pointed, numbering all households along the way. A random number was chosen to identify one of these households as the starting household for the cluster and collection continued on the right-hand side of this starting house until the required number of individuals had been recruited for the sample. If neither household members nor a woman as per the selection criteria, were present at the time of the survey visit, the next closest household was chosen.

A total of 1,115 households from the 19 *tabias* and 30 selected clusters were visited from August to September 2009. The units of analysis for this study were women (aged 15–49 years) who had given at least one live birth during the five years before the survey.

### Study subjects and data collection

A structured questionnaire was prepared in English, based on an existing tool and translated into the local language (Tigrigna) prior to the start of the fieldwork (Additional file 1). To ensure that the questions were clear and could be understood by both the enumerators and the respondents, the questionnaire was pretested and further refined based on the results. The questionnaire collected information on socio-demographic and obstetric characteristics, use of ANC and place of delivery.

Fifteen HEW enumerators who were fluent in the local language and four supervisors with experience in maternal health service provision were selected for data collection. The supervisors were assigned to supervise the data collection process and perform quality checks. Three days of training were given to both the data collectors and the supervisors and were managed by the investigator. The training focused on the quality of the field operation (how to fill in the questionnaire, mock interviews and other practical exercises).

### Ethical clearance

Permission to carry out the study was obtained from the Tigray Regional Health Bureau and the Samre-Saharti District Health Office. Each respondent gave informed verbal consent after being told the purpose and procedures of the study. All responses were kept confidential and anonymous.

### Study variables

Two response variables were created from questions included in the study questionnaire on ANC and place of delivery. ANC use was defined as whether the mother paid at least one visit to the health post during her pregnancy. Place of delivery was classified as home delivery or institutional delivery, the latter including births that took place at a health centre or at a health post. Despite its importance, PNC was not included in this study since this strategy is under developed in Tigray region.

In order to study the influence of explanatory variables on the utilisation of ANC and place of delivery, several predictor variables were selected based on (national and international) literature, national guidelines, field observations and common local practices. The independent variables were categorised as follows. The ages of mothers were grouped as 16–29 years, 30–39 years and 40–49 years. Marital status was classified as married for those who were currently living with their partners, single for those who had never been married, divorced for those currently separated, and widowed for those who had lost their husbands. Respondents’ education was classified as illiterate, grade 1–4, grade 5–8 and grade 9–12 and above. Husbands´ occupation was classified as farmers, and other occupations (which included pay-in-cash jobs such as daily labourers, merchants and governmental employees). Proximity of residence to health facility was defined as the availability or not of a health facility in the village. Parity was grouped as 1–4, 5–7 and 8–11 children. History of obstructed and prolonged labour was defined as whether the mother had reported experience of difficult labour in a previous pregnancy. A question about receiving pregnancy advice or not during ANC visits was also included in the questionnaire.

### Data analysis

Data were collected, compiled and reviewed by the supervisors and then entered into Epi Info software, coded, cleaned, and finally imported into STATA version 10 software for analysis. Univariable logistic regression was carried out between the selected predictor variables and the outcomes (ANC and institutional delivery service utilisation). Those variables which were significant (i.e. with a p value<0.05) in the univariable logistic regression were selected and retained in the multivariable logistic model. Marital status, education, parity, health facility availability in kushet and husband’s occupation were included for the first outcome variable ANC. In addition to the mentioned variables, ANC attendance, ANC advice and difficult/prolonged labour were included in the adjusted models for the delivery care outcome variable. Both variables ‘Attended ANC’ and ‘ANC advice’ were included in the analysis despite potential collinearity (although correlation coefficient of 0.56 was observed in the analysis) because not all women attending ANC received advice and we wanted to capture this dimension. Odds ratios and their 95% confidence intervals (CIs) were calculated.

## Results

### Socio-demographic characteristics

The response rate was 99% (n=1113). Table 1 presents the main socio-demographic characteristics of the participants. Of the total respondents, 880 (79%) were non-educated, 986 (88.6%) were married and 473 (42.5%) were in the young age group, 16–29 years. The mean age of the participants was 30.4 years. Of the total sample, 841 (75.6%) had at least one family member who had attended formal education. Most of the women had experienced either 1–4 (53%) or 5–7 pregnancies (33%). Over half of them (62%) had no health facility in their village and 980 (88%) reported that their husband’s occupation was farming. The average number of live births per women was five-six. Among those who reported having attended schooling, 11% and 6.3% had finished grades 1–4 and 5–8 respectively. Only 3.3% had completed secondary school education. Almost one-fifth (n=228) of the participants reported a history of difficult/prolonged labour from their previous births. The results also showed that 25% of respondents had to walk for an average of one-two hours to reach the health post. The decision to visit a health facility for delivery was made by the mothers themselves in most cases (74%), followed by the extended family (the woman’s mother and father, mother-in-law, elderly relatives and neighbours) (12%), and the husbands and TBAs (4% respectively). The remaining 6% was involved a joint decision made by husbands and wives. The proportion of women who perceived the health facility as a better place to give birth than at home was 63%. Furthermore, around 40% of women said they wanted to give birth at a health facility next time. Women´s preference for being assisted by health workers, mothers and TBAs for their next birth was 50%, 25% and 17%, respectively.

**Table 1:** Women’s characteristics and their frequency and proportion in Saharti-Samre district (N=1113)

**Individual variables**	**Number of women**	**(%)**
Age groups		
16-29	473	43
30-39	482	43
40-50	158	14
Marital status		
Widowed+Single	60	5
Married	986	89
Divorced	67	6
Education		
Illiterate	880	79
1-4 Grade	126	11
5-12 Grade	107	10
Parity		
1-4	593	53
5-7	371	33
8-11	149	13
Husbands´ Occupation		
Farmer	981	88
Government employee	31	3
Daily laborer	50	4
Merchant	51	5
Health facility in village		
No	687	62
Yes	426	38
Total deliveries		
Home	1067	96
Health facility	46	4
Health center	41	3.6
Health post	5	0.5
ANC use		
No	511	46
Yes	602	54

### Antenatal care

The findings of this study showed that 602 women (54%) had received ANC services at a health facility at least once during their last pregnancy. Some of the reasons mentioned for attending ANC were “to know maternal health status” (60.3%), “because of sickness” (31%), and “to know foetal status” (26.4%). Among those who did not attend ANC, the most frequently mentioned reasons were “not feeling sick” (32.7%), “lack of awareness of the benefits” (28.2%), “feeling shame” (16.7%), “workload” (13.4%) and “health facility too far away” (12.5%).

There was an equal proportion of ANC users (23.5%) among the 16–29 years and 30–39 years age groups, whereas ANC use was very low (7%) in the older age group. Nearly half (49%) of those who received ANC were married. Several factors were found to be significant predictors for ANC utilisation (Table 2). Married (OR=2.57, 95% CI: 1.44-4.58) and divorced (OR=2.78, 95% CI: 1.31-5.89) women had a higher probability of visiting ANC services than single and widowed women. With regard to education, mothers with 5–12 years of education (OR=3.18, 95% CI: 1.85-5.47) were more likely to attend ANC than non-educated and grade 1–4 mothers. Proximity of the health facility in the village (OR=1.83, 95% CI: 1.41-2.38) and having husbands with a non-farming occupation (OR=2.26, 95% CI: 1.43-3.58) were also associated with a greater use of ANC.

**Table 2:** Women’s characteristics and its association with attending antenatal care for their recent birth in Saharti-Samre district (n=1113)

**Individual variables**	**Received ANC**		**Odds ratio (95% CI)**	**Adjusted odds ratio (95% CI)**
	**n (%)**			
**Age groups**	Yes	No		
**16-29**	262 (23.5)	211 (18.9)	1	
**30-39**	262 (23.5)	220 (19.8)	0.95 (0.73-1.2)	
**40-50**	78 (7.0)	80 (7.2)	0.78 (0.54-1.12)	
**Marital status**				
**Single+Widowed**	19 (1.7)	41 (3.7)	1	1
**Married**	546 (49.0)	440 (39.5)	2.68 (1.54-4.69)	2.57 (1.44-4.58)
**Divorced**	37 (3.3)	30 (2.7)	2.66 (1.29-5.50)	2.78 (1.31-5.89)
**Education**				
**No education**	452 (40.7)	428 (38.4)	1	1
**1-4 Grade**	64 (5.8)	62 (5.6)	0.98 (0.67-1.42)	0.96 (0.65-1.42)
**5-12 Grade**	86(7.7)	21 (2.4)	4.12 (2.49-6.83)	3.18 (1.85-5.47)
**Parity**				
**1-4**	320 (28.8)	273 (24.5)	1	
**5-7**	202 (18.1)	169 (15.2)	1.02 (0.78-1.31)	1.16 (0.88-1.55)
**8-11**	80 (7.2)	69 (6.2)	0.99 (0.69-1.41)	1.28 (0.87-1.88)
**Health facility in village**				
**No**	325 (29.2)	362 (32.5)	1	1
**Yes**	277 (24.9)	149 (13.4)	2.08 (1.62-2.68)	1.83 (1.41-2.38)
**Husbands Occupation**				
**Farmer**	499 (44.8)	482 (43.3)	1	1
**Others**	103 (9.2)	29 (2.6)	3.42 (2.23-5.27)	2.26 (1.43-3.58)

### Place of delivery

The study showed that institutional delivery service utilisation was very low. Of all the survey participants, only 46 women (4.1%) gave birth at a health facility, 41 of them (3.6%) at a health centre and five (0.5%) at a health post. The most frequently mentioned reasons for delivering at a health facility were “saves mother’s life” (31.2%), “health facility is clean” (30.6%), “bleeding will not occur” (21%), “problem of retained placenta is not encountered if delivered at health facility” (12.5%) and “health facility supports labour” (10%).

The study noted that 95.9% of women were assisted at home: by their mothers (or other elderly women who were relatives or neighbours) for more than half (53.2%); by TBAs (40%), and by HEWs (6.8%). The most frequently mentioned reasons for delivering at home were “easy labour” (64%), “transport problem” (4%), “health facility too far” (4.7%) and “afraid of user fees” (1%).

Women in the younger age group had the highest proportion of institutional delivery use (54%) followed by the middle age group, 30–39 years (39%). Moreover, women with 5–12 years of education were more likely to use institutional delivery than non-educated mothers (OR=2.56, 95% CI: 1.1-6.0). Parity was another important determinant. Women with 8–11 (OR=0.40, 95% CI: 0.11-1.48) and 5–7 children (OR=0.39, 95% CI: 0.17-0.93) were less likely to use institutional delivery than women with less parity.

Receiving health information about ANC during the pregnancy check-up (OR=3.08, 95% CI: 1.21-7.84), history of difficult/prolonged labour (OR=10.0, 95% CI: 4.87-20.6) and husband’s occupations classified as other than farming (OR=3.84, 95% CI: 1.78-8.29) were also strongly associated with the use of institutional delivery (Table 3).

**Table 3:** Women’s characteristics and its association with home delivery for their recent birth in Saharti-Samre district (n=1113)

**Characteristics**	**Place of delivery**		**Odds ratio (95% CI)**	**Adjusted odds ratio (95% CI)**
	**Home n (%)**	**HF n (%)**		
Age groups				
16-29	448 (40.3)	25 (2.2)	1	
30-39	464 (41.7)	18 (1.6)	0.69 (0.37-1.29)	
40-50	155 (13.9)	3 (0.3)	0.35 (0.10-1.16)	
Marital status				
Single+Widowed	55 (4.9)	5 (0.4)	1	
Married	945 (84.9)	41 (3.7)	0.47 (0.18-1.23)	
Divorced	67 (6.0)	0	-	
Education				
No education	854 (76.7)	26 (2.3)	1	1
1-4 Grade	123 (11.1)	3 (0.3)	0.83 (0.25-2.8)	0.72 (0.20-2.55)
5-12 Grade	90 (8.1)	17 (1.5)	6.9 (3.63-13.2)	2.56 (1.08-6.01)
Parity				
1-4	560 (50.3)	33 (3.0)	1	1
5-7	361 (32.4)	10 (0.9)	0.47 (0.23-0.96)	0.39 (0.17-0.93)
8-11	146 (13.1)	3 (0.3)	0.35 (0.11-1.15)	0.40 (0.11-1.48)
Husbands occupation				
Farmer	957 (86)	23 (2.1)	1	1
Other occupations	109 (9.8)	23 (2.1)	8.78 (4.77-16.2)	3.84 (1.78-8.29)
Attended ANC				
No	504 (45.3)	7 (0.6)	1	1
Yes	563 (50.6)	39 (3.5)	4.98 (2.21-11.2)	1.45 (0.48-4.34)
ANC advice				
No	634 (57.0)	12 (1.1)	1	1
Yes	433 (38.9)	34 (3.1)	4.14 (2.1-8.1)	3.08 (1.21-7.84)
Difficult/prolongedlabor*				
No	837 (77.4)	17 (1.6)	1	1
Yes	200 (18.5)	28 (2.5)	6.89 (3.70-12.8)	10.0 (4.87-20.6)
Health facility in village				
No	666 (59.8)	21 (2.0)	1	
Yes	401 (36.0)	25 (2.2)	1.97 (1.1-3.57)	1.59 (0.77-3.27)

* Number of respondents for difficult/prolonged labor is 1082 (1036/46), because of missing reports.

## Discussion

This study showed a moderate coverage of ANC (54%) but very low institutional delivery utilization (4.1%). The factors associated with ANC utilization were marital status, five and more years of education, proximity of health facility to the village, and husband’s occupation other than farmer, whereas for institutional delivery, less parity, five or more years of education, having received ANC advice and history of difficult/prolonged labour were the main associated predictors.

The prevalence of ANC use in this study was similar to other recent national studies [21,29]. It was higher than in the EDHS 2011 (34%), which might indicate some progress, but lower than in the estimated data from the Tigray Health Bureau (THB) (75%) [28]. The prevalence of skilled delivery attendance in rural Tigray is one of the lowest in the world, even when compared with other sub-Saharan African settings [30-32]. Similar data were collected in rural areas of South East Ethiopia (4.3%) [29], although higher prevalence has been observed in other regions (12.1%, 12.3%, 16%, 18.2%) [21,22,26,33] and in reports from the THB (20%). An explanation of these differences might reflect the fact that aggregated data collected by the THB do not show inequalities in ANC, as well as institutional delivery utilisation between different geographical areas: urban better-off areas versus rural areas, such as the one represented in this study, where women face more difficulties in accessing services. On the other hand, although institutional delivery is low in this study, a substantial proportion of women (50%) are in favour of it. This could be for various reasons. It might be due to social-desirability bias, or because women might not have the decision-making power, or that even if they want to have an institutional delivery the barriers are so large that their wish would be beyond their control.

The frequently mentioned reasons for home delivery in this study were “easy birth” (meaning non-complicated birth) and “sudden onset of labour”. Similar findings were described in the EDHS 2011 in which 77% of women from the Tigray region said that the reason for not using health facility delivery was that they perceived it as “not necessary” [20]. Other studies have shown the historical connection between childbirth and the traditions and customs of the community, the influence of religion, and how the strong relationship between female elderly relatives and TBAs plays a major role for women in choosing to give birth at home [23,29,34-36]. For instance, a previous qualitative study from Tigray region revealed a major impact of religion where women relying on God’s will during delivery decided to stay at home. Similarly, TBAs and elderly relatives at the household level strongly influenced women in giving birth at home [36].

Among health facility deliveries, only five women (0.5%) gave birth at the health post. Surprisingly, of those who gave birth at home, only 6.8% were assisted by HEWs. This was an unexpected outcome, since HEWs are expected to provide a clean and safe delivery at the household level. Within the HEP, women are encouraged to call HEWs who are close to their vicinity for home delivery. However, the community might have various reasons for not consulting HEWs. Other studies have, for instance, mentioned the lack of trust in the capacity of HEWs for managing labour and complications and their insignificant role in assisting births [10,23,37-39]. The absence of water and electricity at the health posts would also prevent women from seeking delivery assistance from HEWs [10].

With regard to civil status, married and divorced women were more likely to use ANC than single and widowed women. This finding is supported by both national [24,40] and international studies [34,41]. Women who conceive out of wedlock were possibly less motivated to attend ANC due to community stigmatisation and marginalisation [24,40], and even providers’ discriminatory attitudes [42].

Although the prevalence of ANC in divorced women is small compared to married women , the higher ANC utilisation by divorced women compared to single and widowed women is still an unique finding in our study. A possible explanation might be that divorced women are more empowered and have more autonomy than single and widowed women. Although there is not much published literature on this issue in the context of the study area, if the divorce is initiated from the woman’s side, it might imply a higher level of self-reliance and autonomy in the woman. Other studies have shown that women’s autonomy is a major contributing factor for ANC use and delivery [43].

The analysis of our results highlighted the effect of women’s education. Having five or more years of education was found to be a significant predictor for both ANC use and institutional delivery. This result was consistent with the findings of several studies conducted in different regions of Ethiopia [21-26,33,44] and other low-income countries [32-34,41-44]. Women’s education improves the status of women, enabling them to make the decision to seek health care and to identify danger signs during pregnancy. Furthermore, it increases women’s knowledge on where and how the best health care can be accessed and enhances women’s capability of making autonomous decisions [45].

It becomes clear from this study that the key factor for improving maternal health care and access is women’s education. This is one of the great challenges for a country where more than half (52%) of the female population are non-educated and only 3.4% have reached secondary level [20]. Though Ethiopian women have struggled for equal rights with men and freedom in society, their value is still measured in terms of their position as a mother and wife [45].

Proximity to the health facility is another one of the main factors involved in improving access to maternal health services [42]. The results of this study confirm the advantages of proximity for using ANC services, but not for institutional delivery. In contrast to the findings of our study, studies from Ethiopia have indicated proximity as a determinant predictor for both ANC and institutional delivery utilisation, although mainly for urban residents [25,40]. Previous studies in other African countries have also shown an association between ANC and institutional delivery utilisation and proximity to a health care facility [30,46]. While proximity is obviously an important health care factor regarding accessibility, in our case other aspects seem to play stronger roles.

Husbands´ occupation was associated with both ANC use and institutional delivery. Women who classified their husbands´ occupation as “other than farming” were more likely to use ANC services and institutional delivery than farmers’ wives. This finding was comparable to the results of other studies where women with husbands in non-farming occupations were more likely to use ANC and institutional delivery [34,42].

Parity appears commonly as a major factor responsible for the utilisation of ANC and institutional delivery. Although studies from Ethiopia [47] and elsewhere in Africa [41] have shown an inverse relationship between parity and the use of ANC services, no association was found in our study. The present study revealed that women having less than five children were more likely to use institutional delivery. This result was consistent with other national studies in which the probability of giving birth at health facilities decreases for women with five or more births [24,25,29]. One reason for this relationship could be the limited access to resources and time constraints related to child care and household activities. Another likely reason is that women with more children perceive delivery as a normal process and develop the confidence to give birth at home [32,42].

Women with a previous history of difficult/prolonged labour were almost eight times more likely to use institutional delivery than their counterparts, which is a common finding in the literature [25,42]. Women with a previous history of complications might have had experience of a life-threatening situation. As a result, they might be more motivated to use institutional delivery [25].

Health information sharing is an essential element of ANC. One important component of ANC is to offer information and counsel women about birth preparedness and promote the importance of institutional delivery; therefore, health workers are expected to provide information on danger signs during pregnancy and other benefits of institutional delivery based on the principles of focused ANC [41]. Although the effect of health messages conveyed in an attempt to change women’s attitude to visit a health facility for delivery may be limited, studies have shown the positive effect of receiving ANC advice on encouraging women to use institutional delivery [30,48]. Other local studies support this finding. For example, a qualitative study in rural Ethiopia indicated that mothers who received prenatal education reported feeling better prepared for childbirth [35]. Similarly, in South East Ethiopia, advice during ANC showed a significant association with utilisation of maternal health services [22,29,33]. The findings of the current study were similar. However, the lack of dissemination of key health information during antenatal visits at health facilities can be considered as a missed opportunity that hinders institutional delivery [35]. The results from our study corroborated this reality, where 58% of women did not receive advice about birth preparedness and signs of pregnancy complications during a pregnancy check-up visit at the health facility. Similar constraints were reflected in a Kenyan study, where one-quarter and one-sixth of women were not informed about danger signs during pregnancy and the importance of institutional delivery, respectively [41]. Apparently, the problem of not conveying key information to women could be explained by an excess workload, because the time allocated for information delivery and counseling could be very small when compared to all the HEP components [21]. In our study, no relationship was found between ANC use and institutional delivery attendance. While a positive association might have been expected [22,33], findings similar to ours can be found in the literature [26,49]. Cultural factors and accessibility barriers definitely play an important role in this context.

### Limitations of the study

The data were collected by HEWs who were familiar to the community, which could result in social desirability bias. However, interviewers were well trained to create a climate of trust with the interviewees were aimed at facilitating sincere responses. The questionnaire assessed the use of maternal health services in the five years preceding the survey, which might have introduced recall bias in the use of services. We are also aware that the reasons given for attending/not attending health facilities are retrospective, with the outcome often stated rather than the actual reason. Moreover, the methodological nature of the cross-sectional study design limited the causality inference of the study variables.

## Conclusion

A relatively acceptable utilisation of ANC services but extremely low institutional delivery was observed. The major factors associated to institutional delivery were education, proximity of health facility to the village and receiving advice during ANC. Although numerous factors are known to impede institutional delivery, improving the quality of ANC through the provision of proper counselling and advice to ANC attendants might increase the rate of institutional delivery. 

The HEP needs to be strengthened through a well-functioning and feasible referral system. The allocation of time to different HEP components by HEWs should be discussed to maximise their contribution to the provision of quality delivery services. The current strategy of child delivery at health post level should be carefully reconsidered.

## Competing interests

The authors declare that they have no competing interests.

## Authors’ contribution

YT contributed to the conception, design, data collection, analysis, interpretation and write up of the paper. TG contributed to the data reanalysis, revising the interpretation and preparing the draft of the manuscript. MSS contributed to the conception, providing scientific advices on the design of the study, data analysis and throughout the preparation of the manuscript. IG and KE contributed in revising the interpretation and throughout the preparation of the manuscript. HL contributed in designing, analysis, interpretation and write up of the paper. All authors read and approved the final manuscript.

## Supplementary Material

Additional file 1 Questionnaire on Determinants of ANC and Institutional Delivery Service utilization in Sharti-samre District, Tigray, Ethiopia. Click here for file
