# Supplementary material for: Determinants of antenatal and delivery care utilization in Tigray region, Ethiopia: a cross-sectional study
Source: Int J Equity Health. 2013 May 14;12:30. doi: 10.1186/1475-9276-12-30 (PMC3658893; doi:10.1186/1475-9276-12-30)
Supplement: Additional file 1 — Questionnaire on Determinants of ANC and Institutional Delivery Service utilization in Sharti-samre District, Tigray, Ethiopia. [file 1475-9276-12-30-S1.docx]

Appendix 1

Questionnaire on Determinants of ANC and Institutional Delivery Service utilization in Sharti-samre District, Tigray, Ethiopia

Interviewer name: //Date:Interviewer code: Kushet name: Household code:Kushet code:

**Social demographic data**

1. Respondent’s age in years __________

2. What is your marital status

5. Separated 4. Widowed 3. Single 2.Divorced 1. Married

3. What is your occupation

4. Housewife 3. Merchant 2. Daily labourer 1. Farmer

6.Other (specify) _________5. Governmental employer

4. What is the level of your education

4. 9–12 grade 3. 5–8 grade 2. 1–4 grade 1. Illiterate

5. Higher education

5. What is your religion

3. Protestant 2. Muslim 1. Orthodox

6. What is the main occupation of your husband?

5.Other (specify)__________ 4. Governmental employer 3. Merchant 2. Daily labourer 1. Farmer

7. What is your husband’s level of education?

4. 9–12 grade 3. 5–8 grade 2. 1–4 grade 1. Illiterate

5. Higher education

2.No 8. Do you have any family members who attend formal education? 1. Yes

9. If your answer for question 8 is yes, what is the level of education? (if your answer is no, skip to question number 10)

4. Higher education 3. 9–12 grade 2. 5–8 grade 1. 1–4 grade

10. Have you ever received education about maternal health?

2.No 1. Yes

11. If your answer for question 9 is yes by whom? (if your answer is no skip to question 12)

5.HW 4. CBRHA 3. TBAs 2. CHWs 1. HEWs

7. Other (specify)_____6. Radio

12. Do you have any health facility in your kushet?

3.Don, t know 2. No 1. Yes

13. How far is the nearest health facility from your house by walking in minutes? ____________

14. What type of community health workers do you have in your kushet?

3.CBRHA 2. TTBA 1. CHW

15. Are you involved in the safety net program?

2. No 1. Yes

16. If your answer for question 15 is yes since which year were you involved _______ (if your answer is no skip to qestion number 17)

17. What is the monthly income of your family? _______________

**Obstetric History**

18. What is the number of total pregnancies in lifetime?

3. Eight- eleven 2. Five-seven 1. One-four

19. What is the number of pregnancies in the last five years?

3. Three 2. Two 1. One

20. What is the number of children under five in your family?

3.three 2. Two 1. One

21. What is the total number of family members in your household?

4. Eight-eleven 3. Five-seven 2.Two - four 1. One-three

22. Do you have history ofmissed/terminated pregnancy?

2. No 1. Yes

23. If your answer is yes for question 22 how many times? (if your answer is no skip to question number 24)

4. More than three 3. Three 2. Two 1. One

24. Have you ever gave birth for a dead fetus?

2. No 1. Yes

25. If your answer for question 24 is yes ) how many times? (if your answer is no skip to question 26)

4.More than three 3.Three 2.Two 1. One

26. Did you attend ANC for your recent pregnancy?

2.No 1. Yes

27. If your answer for question 26 is yes at which pregnancy weeks did you start? ____ (if your answer is no skip to question 28)

28. If your answer for question 26 is yes how many visits did you attend?______________

29. If your answer for question 26 is yes, what are the reasons for attending ANC?

7.Other (specify)____________ 6. To know the status of my fetus 5.To know the status of my health 4. My Husband encouraged me 3. Good service of health facility 2.Health facility was close 1. I was sick

30. If your answer for question 26 is yes, during your visit, did you receive any advice where to deliver? (if your answer is no skip to question 32)

No 1. Yes

31. If your answer for question 26 is no, what are the reasons for not attending ANC?

9. Other (specify)________ 8. I Don, t know the benefit 7.I Feel shame of being pregnant 6.HWs poor approach 5. Afraid of service fee 4. My husband refused 3.Health facility was far 2. I had work load 1. I had no health problem

**History of the Recent Delivery**

32. What was the year of birth for your recent baby_______________

33. Where was the place of birth for your recent baby

3. Health center 2 Health post 1. At home

34. If your answer for question 33 is at home, who assisted you

4. Neighbor 3. TBA 2. Mother –in-law 1. Mother

6. 0thers specify_______5. HEW

35. If your answer for question 33 is at health facility, who assisted you?

4. Health officer 3. Midwife 2. Nurse 1. HEW

5. Don’t remember

36. If your answer for question number 33 is at home, why?

3.Health facility (HF) far away 2. Transport problem 1. I had easy labor

12. Other (specify)_________ 11. I was sick 10. I Don’t think it was important to use HF 9. Poor skill of health workers at HF 8. Saint Marry helps me 7. Felt ashamed at HF 6. Poor service at HF 5. Afraid of user fee at HF 4. Husband refused HF

37. If you gave birth at health facility for the recent baby, why?

3. Health facilitywas close 2. The service is provided free 1. I was sick

6. The HF saves mother’s life 5. Family allowed 4. Good service

7. Received health education about HF

8. Other (specify)__________

38. Who decided where you gave birth?

4. Mother 3. Both of us 2.My husband 1. Myself

8. HW 7. Neighbor 6. TTBA 5. Mother –in-law

11. 0thers specify______ 10.TBA 9. Father

39. Do you think there is a difference between giving birth at home compared to at a health facility?

3. Don’t know 2. No1. Yes

40. If you think health facility is better compared to giving birth at home (question 39) why?

7. Other (specify)__________ 6.HF Shorten labor 5.HF Save children’s lives 4. No bleeding 3. Problem of retained placenta is not encountered if delivered at a HF 2. HF Save mothers’ lives 1. HF is clean

41. If you think the home is better for delivery compared to health facility (question 39) why?

3.If no bleeding, the home is better 2. No cost 1. No need of transport

5.Other (specify)______________ 4. There is privacy

42. Do you have any history of difficult/prolonged labor labor?

2. No 1. Yes

43. If your answer is yes for question (42) which type of complication? (if your answer is no skip to qes 44)

4. Abnormal presentation of the fetus 3.Prolonged labor 2. Retained placenta 1. Bleeding

6.Other (specify)____5. Dead fetus in my womb

44. Where do you prefer to give birth next time?

2.At a health facility 1. At home

45. If you became pregnant for your next delivery, by whom do you prefer to be assisted?

\ 6. Others specify_______ 5. HW 4. Neighbor 3. TBA 2. Mother –in- law 1. Mother

46. Where do your husband prefer you to give birth next time?

2. At a health facility 1. At home
